# Talker and accent familiarity yield advantages for voice identity perception: A voice sorting study

**DOI:** 10.3758/s13421-022-01296-0

**Published:** 2022-03-10

**Authors:** Sheriff Njie, Nadine Lavan, Carolyn McGettigan

**Affiliations:** 1 Department of Speech, Hearing and Phonetic Sciences, University College London, Chandler House 2 Wakefield Street, London, WC1N 1PF UK; 2 Department of Psychology, School of Biological and Chemical Sciences, Queen Mary University of London, Mile End Road, London, E1 4NS UK

**Keywords:** Voice identity, Accent, Familiarity, Within-person variability

## Abstract

In the current study, we examine and compare the effects of talker and accent familiarity in the context of a voice identity sorting task, using naturally varying voice recording samples from the TV show *Derry Girls*. Voice samples were thus all spoken with a regional accent of UK/Irish English (from [London]derry). We tested four listener groups: Listeners were either familiar or unfamiliar with the TV show (and therefore the talker identities) and were either highly familiar or relatively less familiar with Northern Irish accents. Both talker and accent familiarity significantly improved accuracy of voice identity sorting performance. However, the talker familiarity benefits were overall larger, and more consistent. We discuss the results in light of a possible hierarchy of familiarity effects and argue that our findings may provide additional evidence for interactions of speech and identity processing pathways in voice identity perception. We also identify some key limitations in the current work and provide suggestions for future studies to address these.

*Orla: “Why’s he making that funny noise?”*

*Michelle: “He’s English Orla, that’s the way they talk.”*

*Derry Girls*

Human listeners can perceive identity-related information from voices, but the accuracy of performance on voice identity perception tasks depends on many factors related to the properties of the stimuli used, and the characteristics of the listeners (Kreiman & Sidtis, 2011; Mathias & Von Kriegstein, 2014). In terms of listener characteristics affecting voice identity perception, it has been reported that, broadly speaking, familiarity with aspects of the stimuli used is advantageous.

Effects of familiarity on voice identity perception have most frequently been tested by contrasting listeners that are either familiar or unfamiliar with the voices used (Lavan, Burston, & Garrido, 2019a; Lavan, Burston, Ladwa, et al., 2019b; Lavan, Kreitewolf, et al., 2021b; Lavan et al., 2020; Lavan et al., 2016; Stevenage et al., 2020). These studies typically use voice identity discrimination or voice identity sorting paradigms, allowing familiar and unfamiliar listeners’ performance to be compared directly on the same task. They have furthermore focused on the effects of talker familiarity in the context of voice stimuli that include within-talker variability. Within-talker variability describes the observation that the sound of a single person’s voice can change dramatically, depending on the speaking situation: People change the sound of their voice to best convey their intentions and feelings and to adapt to their audience and acoustic environment (Lavan, Burton, Scott, & McGettigan, 2019c). The results of this body of work on talker familiarity effects have converged on two key findings: (1) within-talker variability generally presents challenges to voice identity perception, but (2) being familiar with a talker enables listeners to largely overcome these challenges, resulting in more accurate identity perception.

Specifically, in voice identity sorting studies, listeners—who can be familiar or unfamiliar with the talkers—are presented with a limited number of naturally varying voice recordings embedded on a drag-and-drop interface (J. Johnson et al., 2020; Lavan, Burston, & Garrido, 2019a; Lavan, Burston, Ladwa, et al., 2019b; Lavan, Collins, & Miah, 2021a; Lavan et al., 2020; Lavan, Smith, & McGettigan, 2022; Stevenage et al., 2020). Such naturally varying voice recordings include different speaking situations, across which properties such as the emotional content (e.g., happy vs. angry) or the speaking style (e.g., formal vs. casual; high vs. low effort) can vary naturally. The voice recordings are usually sampled from two to three identities, such that each voice is represented by a number of naturally varying recordings while maintaining a manageable overall number of sounds to be sorted. Participants are asked to listen to the voice recordings and sort them into clusters by identity. The pattern of results that emerges across all studies is that listeners who are not familiar with the talkers perceive several more identities than are actually present, as indicated by a larger number of clusters. When examining how these different clusters are formed, it becomes apparent that the unfamiliar listeners perceive variable voice recordings from the same talker as multiple different talkers (see J. Johnson et al., 2020; Lavan, Burston, & Garrido, 2019a; Lavan, Burston, Ladwa, et al., 2019b; Lavan, Collins, & Miah, 2021a; Lavan, Smith, & McGettigan, 2022; Stevenage et al., 2020). Unfamiliar listeners thus frequently fail to “tell together” variable voice recordings, misinterpreting within-talker variability as between-talker variability. Crucially, if listeners are familiar with the talkers, accuracy improves drastically: While performance is not always perfect, familiar listeners tend to accurately interpret the within-talker variability as such, and therefore perceive variable examples of a talker’s voice as coming from the same identity (Lavan, Burston, & Garrido, 2019a; Lavan, Burston, Ladwa, et al., 2019b; Stevenage et al., 2020). Speaker discrimination studies contrasting familiar and unfamiliar listeners furthermore corroborate these performance benefits when participants are familiar with the talkers (Lavan et al., 2016; Lavan, Kreitewolf, et al., 2021b).

Familiarity with the talkers in a task is, however, only one type of familiarity that may benefit listeners when making identity judgements. Other studies have shown that being familiar with the language used by the talkers producing the stimuli can result in more accurate identity perception, a finding referred to as the language familiarity effect: In their review, Perrachione (2018) provides a detailed examination of these language familiarity effects, reporting that studies using a variety of voice identity discrimination (i.e., same–different identity judgements on pairs of sounds), line-up (i.e., determining the presence or absence of a previously heard voice in an array), or recognition/identification (e.g., categorizing a voice as “old/known” or “new/unknown”; labelling a speaker by name) tasks consistently report better performance when the language used in the voice recordings is the listeners’ native language. However, language familiarity advantages do not always depend on understanding what is being said: Some studies have found that mere exposure to foreign languages could give benefits to performance on voice identity tasks, even when the listeners had little/no competence (e.g., E. K. Johnson et al., 2011; Orena et al., 2015; though see Perrachione & Wong, 2007). Zarate et al. (2015) similarly report that language familiarity advantages appear to work along a gradient, where knowledge of talker identity can transfer to other unfamiliar languages, if those languages contain similar phonological information (e.g., English and German).

In the current study, however, our focus is on the effect of accent familiarity in voice identity processing tasks. Advantages in talker identity perception due to accents have also been reported: Goggin et al. (1991) found that for monolingual English speakers, performance in voice identity line-up tasks is less accurate for Spanish-accented English compared with American-accented English. A number of studies have furthermore investigated whether familiarity with a regional accent within a listeners’ native language may also aid identity perception. A study focussing on forensic applications of talker identity perception (Stevenage et al., 2012) reported that performance is indeed more accurate in a voice line-up task when the target talker speaks with the listeners’ own accent (English vs. Scottish). Kerstholt et al. (2006) furthermore reported similar effects of more accurate voice-line up identification for a familiar (standard) Dutch accent versus a less familiar regionally and socially marked accent from The Hague. Braun et al. (2018) even report some accent familiarity advantages at a sub-regional level for voice line-ups, contrasting different accents from the North-East of England (Newcastle, Sunderland, and Middlesbrough). In this study, misses (that is, discounting the target speaker as a foil voice) were significantly lower for target talkers with the listeners’ own specific accent, while performance for hits and false alarms was comparable across own and other accents. In contrast to the aforementioned studies, Johnson et al. (2018) reported no own-accent advantage in talker line-up tasks when contrasting American and Australian English. Beyond regional accents, sociocultural accents (e.g., Black American English vs. White American English) can elicit own-accent advantages (Perrachione et al., 2010. Overall, the published work suggests that there is substantial evidence for a language familiarity effect when contrasting voice identity perception in the listeners’ native vs. a foreign language, as well as own-accent (defined primarily via regional or sociocultural criteria) advantages across different experimental tasks.

Several explanations have been proposed for when and why both talker familiarity and language or accent familiarity advantages arise for voice identity perception. For identity perception, it has been proposed that familiar listeners can access a talker-specific representation of the voices in question (Lavner et al., 2001; Maguinness et al., 2018), which may also include information about how the voice of a familiar identity can vary (Lavan, Burton, Scott, & McGettigan, 2019c). Such a representation may then enable familiar listeners to cope with the within-talker variability of familiar voices and arrive at an accurate percept for the purposes of identification, for example. Unfamiliar listeners, however, lack such a talker-specific representation. Therefore, they must rely on more general knowledge of how voices can vary when deciding whether two variable recordings of voices were produced by the same speaker or two different speakers (voice identity discrimination being the most typical method for measuring voice identity perception in unfamiliar listeners: Lavan, Burton, Scott, & McGettigan, 2019c; Lavan et al., 2016). For language familiarity benefits, the key explanations ascribe benefits to primarily being familiar with the phonetic, phonological, and phonotactic features of the language (see Perrachione, 2018 for a recent review; see Fecher and Johnson (2018), Fleming et al. (2014), Johnson et al. (2011), Johnson et al., (2018) for studies specifically arguing for a role for phonological information). An alternative approach suggests that the ability to process the linguistic information in the stimuli—at the word level and above—is *additionally* contributing to familiarity benefits in voice identity perception at the acoustic-phonetic and phonological levels (again, see Perrachione, 2018, for a review; Bregman & Creel, 2014; Goggin et al., 1991; Perrachione & Wong, 2007; Perrachione et al., 2011, for relevant empirical findings).

What these explanations have in common is that they share the proposal that familiarity enables listeners to process information in vocal signals that unfamiliar listeners process less effectively. Familiarity with a talker allows the listener to access information about the possible variability in the sound of a specific talker, while language or accent familiarity allows the listener to—potentially additionally—perceive idiosyncrasies in the speech of a talker (e.g., a characteristic way of pronouncing certain words) that would not be salient to a listener lacking relevant accent/language knowledge. Intriguingly, the reported advantages for accent and language familiarity during identity processing, alongside the mechanistic explanations, are partially at odds with a classic model of voice perception (Belin et al., 2004). In that model, the processing of speech- and identity-related information can under certain circumstances interact, but these are ultimately considered to be largely independent. Furthermore, only the voice identity pathway of the model feeds directly into “Person Identity Nodes” (Belin et al., 2004).

In the current study, we investigated the effects of familiarity with talkers and with their accent on performance in a voice identity sorting task. To our knowledge, talker familiarity and language familiarity have never been jointly investigated within the same experimental task. Similarly, studies of accent familiarity effects on voice identity perception have to date tended not to employ naturally varying stimuli, nor have they used identity sorting tasks. However, it is worth noting that, complementary to the voice identity sorting paradigm used here, similarly structured “free classification” tasks have been used to measure naturalistic categorization of multiple foreign and regional accents, with reported effects of listeners’ accent familiarity on their performance (e.g., Clopper & Pisoni, 2006).

Here, we used naturally varying voice recordings from two characters from the TV show *Derry Girls*. Stimuli in our study thus featured a pronounced Northern Irish accent, specifically that of (London)derry. We tested four groups of listeners: To manipulate talker familiarity, we recruited listeners who were either familiar or unfamiliar with the TV show (and thus the identities). To manipulate accent familiarity, we recruited listeners who were either from Northern Ireland (and thus highly familiar with the accent) or from the South East and East of England (and thus less familiar with the accent). Using a between-subjects design with only one stimulus set avoids the limitations of comparing multiple stimulus sets that may be difficult to match (e.g., on talker acoustic characteristics or perceived social status of the accent). We note at this stage that in the experimental design described above, talker and accent familiarity are partially overlapping. For example, listeners from the East of England who have watched the TV show *Derry Girls* have not only become familiar with the different talkers but will also have become more familiar with the Northern Irish accents that feature in the show. Our results therefore need to be interpreted in light of these partially overlapping types of familiarity.

Using this design, we were able to, for the first time, (1) simultaneously measure and compare the effects of both talker and accent familiarity, (2) within a single task, and (3) using a common set of naturally varying stimuli. Based on previous research on the effect of familiarity with the talker, we predicted that listeners who are familiar with the talkers will show significantly higher accuracy in voice identity sorting than listeners who are not familiar with the talkers. We furthermore predicted that accent familiarity would have significant effects on voice identity sorting, such that accuracy would be higher for listeners who are familiar with the accent. Finally, we predicted that the effects of accent familiarity may be smaller than the effects of talker familiarity: Being able to recognize and identify individual talkers is assistive to voice identity sorting performance, but this ability depends essentially on talker familiarity—in contrast, accent familiarity may assist talker discrimination for an unfamiliar listener, but by definition a listener with no talker familiarity cannot recognize or identify that talker.

## Methods

### Participants

In total 165 participants were tested for this study. All participants were between the ages of 18 and 40 years, reported English as their first language (knowledge of other languages was not recorded), and did not have any self-reported hearing difficulties or language processing difficulties. We recruited four groups of participants via the online recruitment platform Prolific.co: The groups included participants who were either familiar or unfamiliar with the TV show *Derry Girls* and participants who were either highly familiar with Northern Irish accents or less familiar with Northern Irish accents. Familiarity with the TV show was established at the point of recruitment rather than post hoc*—*this was done to minimize data wastage in achieving balanced group sizes (assuming the majority of recruited participants would be unfamiliar with the show) and to homogenize participant expectations across the groups about the nature of the task stimuli. In the study advertisement we also informed participants that they would need to use a desktop computer, have the ability to play sounds, and have access to PowerPoint in order to complete the experiment. Although participants were not required to use headphones specifically, within the experiment they were recommended to complete the main task in a quiet environment.

Familiarity with Northern Irish accents was primarily established using preselection criteria in Prolific: Using these criteria, familiar participants were defined as those who had spent the first 18 years of their lives mostly in Northern Ireland and were currently still living there. Participants who were deemed to be less familiar with the Northern Irish accent were recruited based on having spent most of the first 18 years of their lives in England and currently living in either the East or South East of England. As a secondary assessment, accent familiarity was assessed via self-report by asking participants to rate their familiarity with a range of UK regional accents (e.g., Welsh, Cockney, Northern Irish) on a scale form 1 (*not familiar at all*) to 10 (*very familiar*). A range of accents was included to reduce the prominence of the rating of the Northern Irish accent in this questionnaire, although it was our only measure of interest. Familiarity with the TV show was established via self-report at the outset of the task (see Procedure).

From the sample, eight participants were excluded because they failed to accurately complete a vigilance check (sorting two identical recordings of a computer-generated male voice into an independent cluster; see Materials and Procedure). Nine further participants were excluded due to having some preexisting familiarity with the talkers—these participants reported to not have watched the TV show but in the debrief questionnaire (see Procedure) reported to have recognized one or both talkers from other TV shows or media coverage. Nineteen participants who had reported having watched the TV show *Derry Girls* were excluded because they reported having watched less than one season of the show, and as such were considered to be insufficiently familiar with the characters. Finally, three participants were excluded since they formed only one cluster after spending a short amount of time on the sorting task*—*this pattern of responses had not been observed in any of the previous identity sorting tasks run in our research group and was thus considered to be sufficiently anomalous to warrant the exclusion of these participants.

After these exclusions, data from 126 participants remained. There were 32 participants in the group that was familiar with both the talkers and the accent (henceforth Talker_HI_Accent_HI_; Mean age = 25.5 years, *SD* = 5.9; 24 female), 29 participants in the group that was familiar with the talkers but less familiar with the accent (Talker_HI_Accent_LO_; Mean age = 28.1 years, *SD* = 5.9; 14 female), 32 participants in the group that was familiar with the accent but unfamiliar with the talkers (Talker_LO_Accent_HI_; Mean age = 27.2 years, *SD* = 4.8; 11 female), and 33 participants in the group that was familiar with neither the talkers nor the accent (Talker_LO_Accent_LO_; Mean age = 27.6 years, *SD* = 6.1, 24 female). Participants in the groups that were less familiar with Northern Irish accents on average rated their familiarity with Northern Irish accents at 3.66 out of 10 (2.70 for listeners unfamiliar with the talkers vs. 4.79 for listeners familiar with the talkers). Participants from Northern Ireland rated their familiarity with Northern Irish accents on average at 9.63 out of 10 (9.34 for listeners unfamiliar with the talkers vs. 9.91 for listeners familiar with the talkers). The study was run 2.5 years after the first season of the TV show was first broadcast, and just over 1 year after the second season had been first broadcast. The study was approved by the ethics committee of UCL’s Division of Psychology and Language Sciences (approval code: SHaPS-2019-CM-030). This sample size was broadly matched to the sample sizes for previous voice and face sorting studies (e.g., Jenkins et al., 2011; Lavan, Burston, & Garrido, 2019a; Stevenage et al., 2020). This study was not preregistered. All data and materials are available from the authors upon reasonable request.

### Materials

#### Derry Girls and the accent(s) of (London)derry

The TV show *Derry Girls* was chosen for the purpose of this study as it is set in Northern Ireland, featuring a local accent of (London)derry. In general, this is an accent that is not heard frequently in Great Britain and is very rarely heard in broadcast media. In terms of its phonological properties, the accent can deviate from the Standard Southern British English accent on several vowels (e.g., centering of the KIT vowel to [ɪ̈], fronted [ae] or [a] in BATH, [ɘʉ] for MOUTH) and consonants (e.g., word initial palatisation of /k/ making “car” into [kjaɹ], rhotic pronunciation of intervocalic [ɹ]) (McCafferty, 2014). Additionally, high rising intonation in declarative utterances (i.e., higher pitch toward the end of a statement) is a salient feature of this, and other, Northern Irish accents. As McCafferty (2014) notes, the phonological properties of (London)derry speech are affected by class and ethnicity (i.e., Catholic or Protestant). In *Derry Girls*, the majority of characters with speaking roles are the pupils and teachers of a Catholic convent school and their families and include a mixture of working- and middle-class presentations. The characters chosen for the current study—Erin Quinn and Michelle Mallon*—*are both portrayed as Catholic and ostensibly from working class families.

Although this (London)derry English may also exhibit particular diagnostic lexical and syntactic variations, the selected stimuli for the current experiment generally followed standard English with two notable exceptions: “Catch yourself on” (meaning something akin to “get a grip”), and “fella” to refer to a young man.

### Task stimuli

In the current voice sorting task, we used 15 naturally varying voice recordings from each of the *Derry Girls* characters Erin Quinn and Michelle Mallon. The actors playing the two characters (Saoirse-Monica Jackson as Erin Quinn and Jamie-Lee O’Donnell as Michelle Mallon) are both from Derry themselves, and thus were speaking/acting in their native accent. This was an important aspect of their selection, as was their matched apparent gender (female) and their similar ages (Jackson born 1993; O’Donnell born 1992). The final stimulus set indicated close acoustic similarity between the two talkers in fundamental frequency (F0; Erin: Mean = 254.6 Hz, *SD* = 52.8 Hz; Michelle: Mean = 247.1 Hz, *SD* = 43.5 Hz) and in F0 variability (Erin: Mean = 48.6 Hz, *SD* = 27.0 Hz; Michelle: Mean = 44.7 Hz, *SD* = 17.1 Hz).

Recordings were sampled from across different scenes, speaking situations, and speaking environments, thus varying in an unconstrained manner in their linguistic content, speaking style, emotional content, and intention conveyed, among other factors. This was done to sample as much of the natural within-person variability of each identity's voice as possible. Each voice recording included a full meaningful utterance (such as “We had a few questions about the British Empire”), and included as little background noise as possible, with no other voices being audible. Recordings were furthermore selected to be non-diagnostic, avoiding catchphrases for what were considered to be particularly memorable scenes or lines. All experimental stimuli were RMS normalized and the average duration of these voice recordings was 2.74 seconds (SD = 0.58). We also generated a sound clip of a male voice via a text-to-speech synthesizer (“The violin is a beautiful instrument but tough to learn”), which was used in a vigilance check (see Procedure).

These voice recordings were embedded in a Microsoft PowerPoint (Microsoft Corporation, Redmond, WA) slide, with each clip being represented by a numbered box. The numbered boxes were distributed across the slide in a random pattern with no clear clusters being apparent from the outset. The computer-generated voice clip was added twice to the PowerPoint slide, forming the basis of the vigilance task. Participants were expected to readily spot the two duplicate recordings of a male voice and sort them into a cluster of their own. If a participant did not correctly sort these two recordings into a separate cluster they were excluded from the data analysis.

### Procedure

The experiment was implemented using the Qualtrics survey software (Qualtrics, Provo, UT). Participants were first presented with an information sheet and gave their informed consent to take part in the study. Participants then stated whether they had watched the TV show *Derry Girls* or not. They were then asked to download the PowerPoint slide including the voice recordings. Participants were informed that this PowerPoint slide included 32 short recordings of voices, linked to numbered boxes (see Materials). They were then asked to listen to these voice recordings and sort them by perceived identity into clusters, such that each cluster only included recordings produced by the same person. Participants could play the voice recordings in whichever order they chose and could replay recordings as many times as they considered necessary. Participants were unaware that the true number of test identities included in the study was in fact two (plus the third, male voice used for the vigilance check). Sorting was achieved via participants dragging and dropping the boxes on the PowerPoint slide. Participants were shown 3 examples that illustrated what a sorted PowerPoint slide could look like. These depicted variable numbers of clusters: importantly, participants were told not be led by the number of clusters shown in these examples. Finally, to minimize ambiguities with regard to which sound files were included in which clusters, participants were asked to circle each cluster.

After participants had completed the sorting task to their satisfaction, they were asked to upload the sorted slide to a website supporting anonymous file sharing. All participants then completed a debrief questionnaire in which they indicated whether they recognized any of the voices present and were asked to describe their strategy for completing the task. For the Talker_HI_Accent_HI_ and Talker_HI_Accent_LO_ groups, who reported to be familiar with the TV show *Derry Girls*, participants were asked which seasons of *Derry Girls* they had watched (“No full season,” “At least one season,” “Both seasons”; see Participants for details of exclusions). They were also asked how many of the specific voice recordings they may have remembered from the show (with the options 0–5, 6–10 and 10+). Due to an error, data for this question were not collected for all familiar participants. However, an inspection of the data that were collected indicated that the distribution of responses was similar for these two groups. We note that this lack of insight into how many stimuli participants perceived to have remembered from watching the TV show potentially presents a confound. We discuss this issue at length in the Discussion section. The experiment took around 20 minutes to complete, and participants were reimbursed for their time at a rate of £7.50 per hour.

### Data processing and statistical analysis

Three key task performance measures were extracted: (1) number of clusters formed, (2) a “telling together” score, and (3) a “telling apart” score. The computer-generated voice recordings associated with the vigilance task were excluded at this stage of the data processing.

**Fig. 1:**
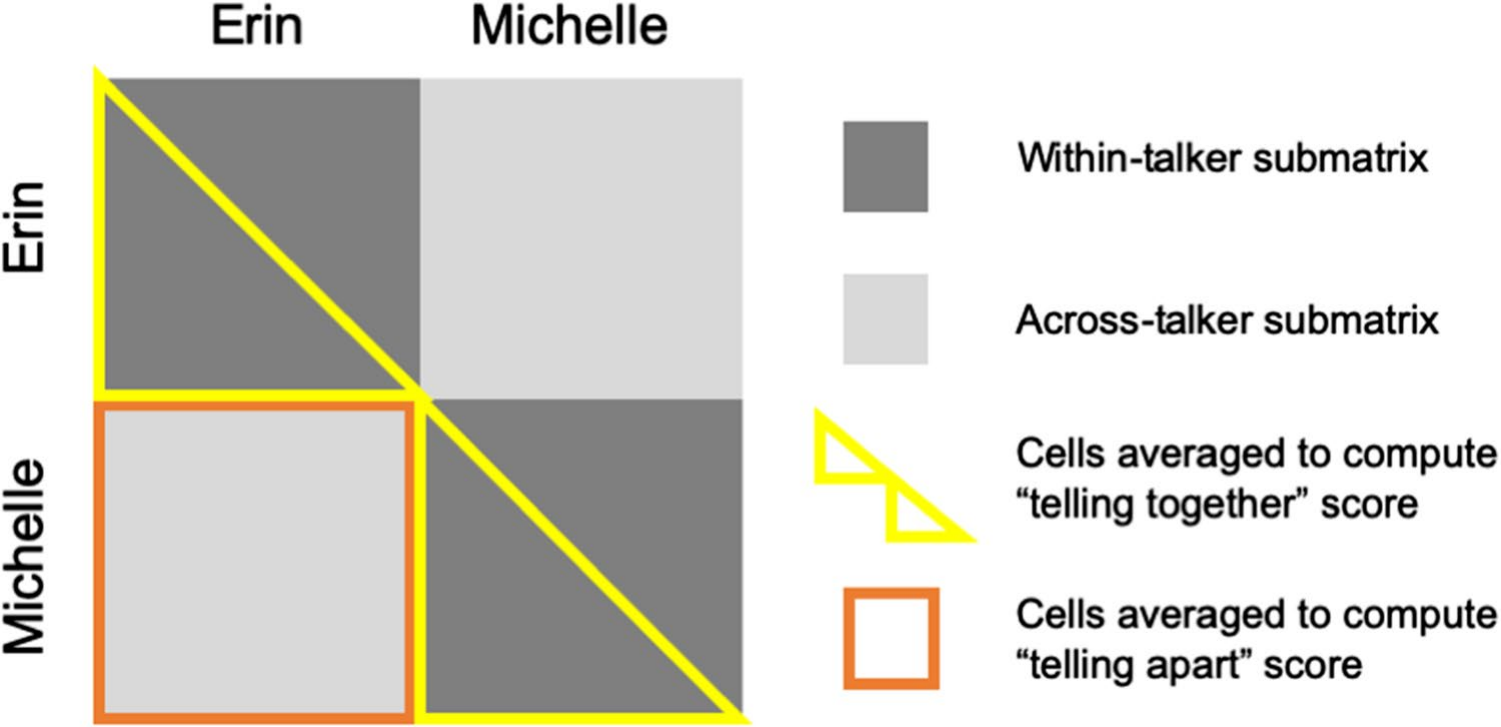
Schematic of a voice identity sorting response matrix, indicating the regions that are averaged to obtain “telling together” and “telling apart” scores

1. The number of clusters corresponds to the number of perceived talkers. With two talkers being the correct answer, increasingly worse performance is indexed by the degree to which the number of clusters exceeds 2. Clusters were counted directly from the sorted PowerPoint slide.
2. The “telling together” score represents listeners’ ability to accurately perceive several variable recordings of a talker as the same identity: To calculate this score, we first created a response matrix in which each cell represents one of all the possible pairwise combinations of the voice recordings. Within this matrix, a pair of stimuli from the same talker was coded as 1 if sorted into the same cluster or 0 if sorted into different clusters. The “telling together” score is computed by taking the average of all unique pairs that veridically included the same talker (see Fig. 1; see also Lavan, Burston, & Garrido, 2019a). If a participant sorts all voice recordings correctly, the “telling together” score is 1. However, if the participant splits voice recordings from the same talker into multiple clusters, the “telling together” score decreases toward 0, indicating worse performance.
3. The “telling apart” score represents listeners’ ability to accurately perceive variable recordings of different talkers as being separate identities. This score is derived from the same response matrix as the “telling together” score (i.e., where 1 = sorted into the same cluster; 0 = sorted into different clusters). For the “telling apart” score, however, the 1s and 0s are averaged across all unique pairs that veridically included the two different talkers (Fig. 1). If a participant never mixes voice recordings of different talkers within the same cluster, the “telling apart” score is 0. If the participant mixes recordings from different talkers within the same clusters, the “telling apart” score increases toward 1, indicating worse performance.

Data were not normally distributed for some of the dependent measures. We therefore used pairwise Mann–Whitney tests to assess the effects of talker and accent familiarity. These tests were implemented in the *coin* package (`Hothorn et al., 2006) in *R*. Statistical significance of tests was determined using an adjusted alpha of .0125, following Bonferroni correction for four pairwise comparisons within each dependent variable (Two comparisons of Talker Familiarity: (1) Talker_HI_Accent_HI_ vs. Talker_LO_Accent_HI_ and (2) Talker_HI_Accent_LO_ vs. Talker_LO_Accent_LO_; Two comparisons of Accent Familiarity: (1) Talker_HI_Accent_HI_ vs. Talker_HI_Accent_LO_ and (2) Talker_LO_Accent_HI_ vs. Talker_LO_Accent_LO_).

The effect size *r* for each test was computed in the *rstatix* package (Kassambara, 2020) in *R*. Effect sizes between 0.1 and 0.3 are considered to be small, 0.3 and 0.5 are considered as medium, and effect sizes over 0.5 are considered to be large. To test our hypothesis that effects of talker familiarity should overall be larger than effects of accents, we qualitatively compared and interpreted these effect sizes (see Perrachione, 2018, for a similar approach).

## Results

### Effects of talker familiarity

**Fig. 2:**
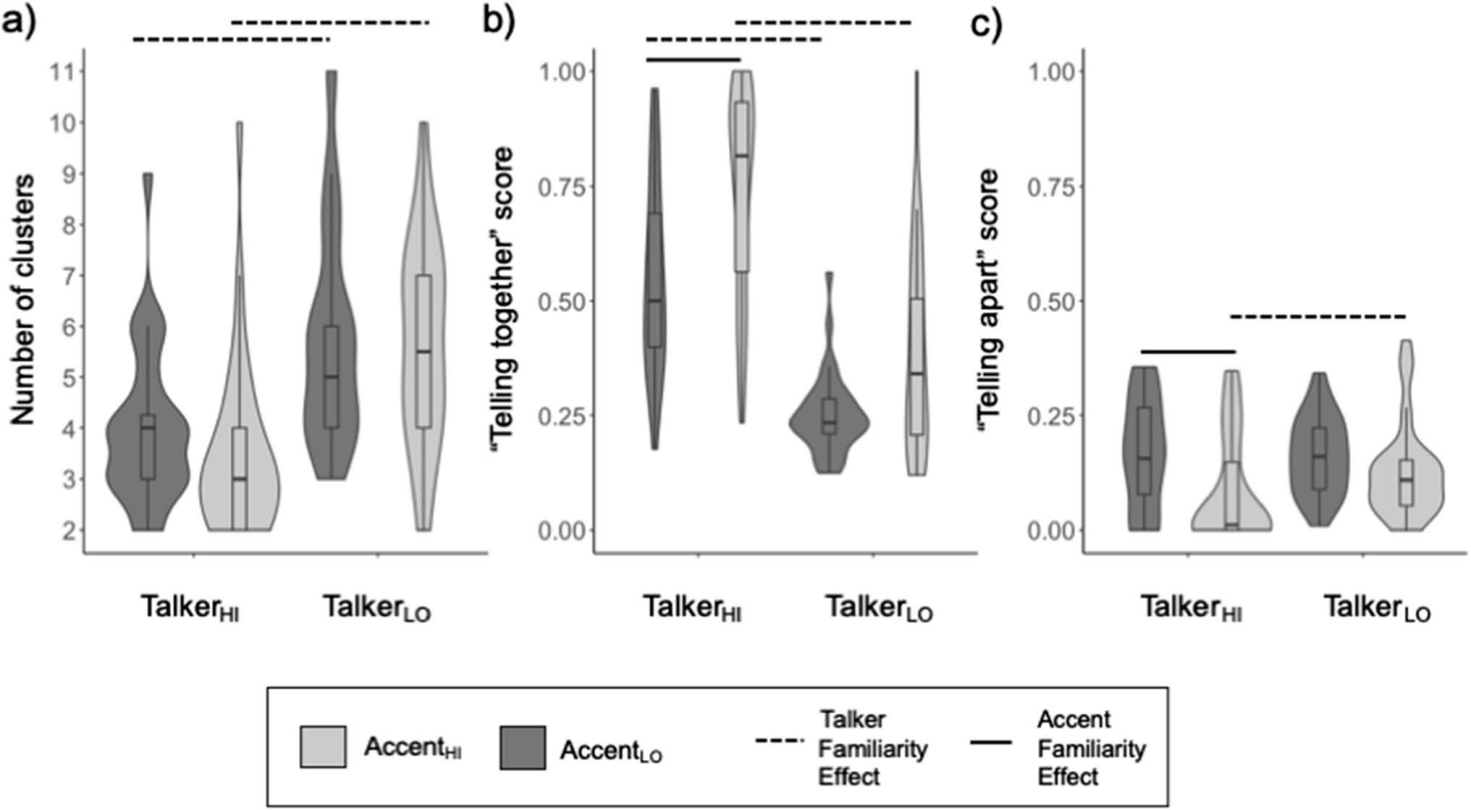
Performance on the voice sorting tasks, plotted by listener group. Lines above the violin plots indicate significant Bonferroni-corrected pairwise comparisons (*p* < .0125). For a description of how the “telling together” and “telling apart” scores were computed, see the main text and Fig. 1

**Fig. 3:**
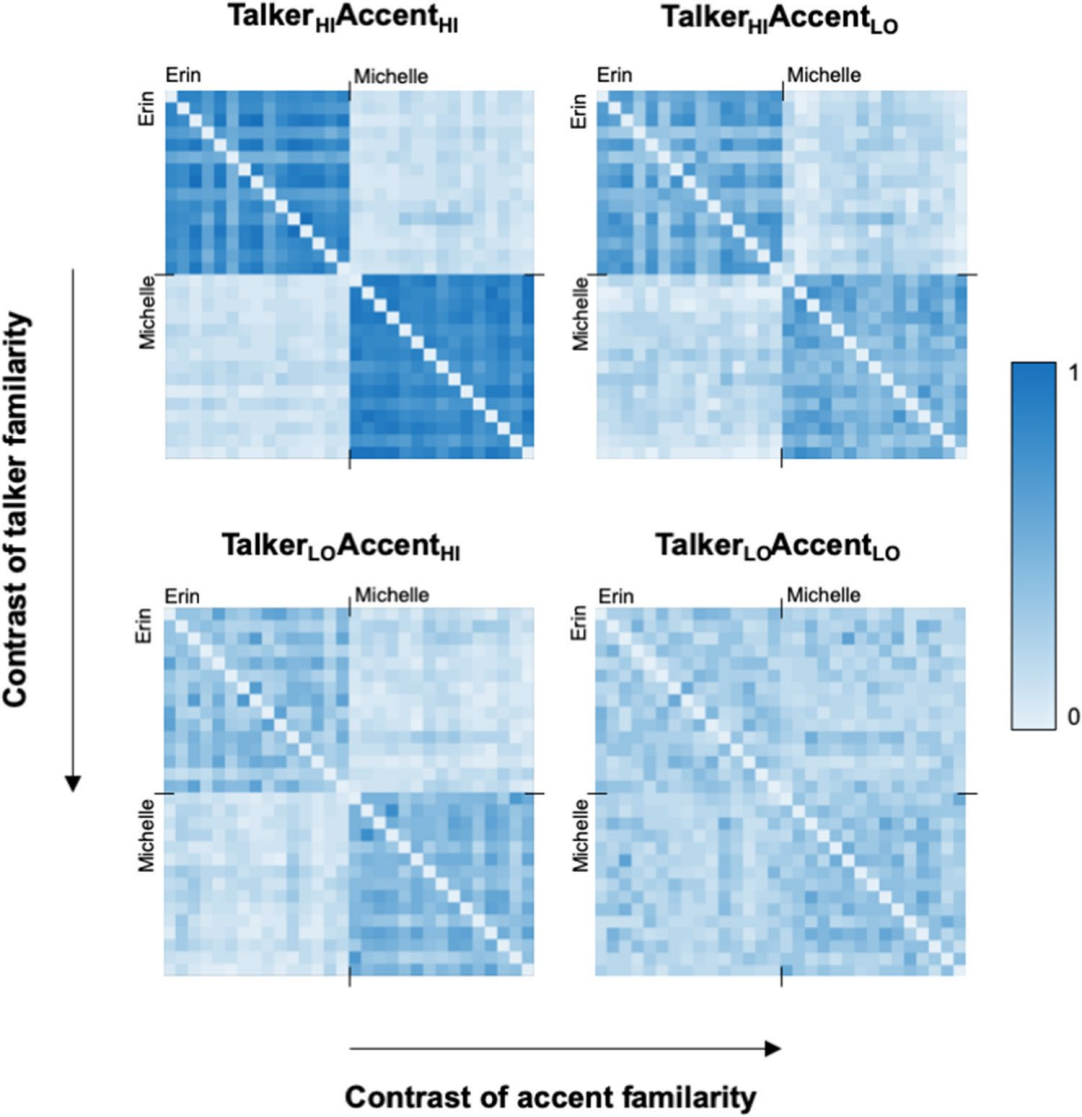
30 × 30 response matrices visualizing mean identity sorting behaviour in each of the four listener groups. Each square represents a pair of voice recordings, where the colour coding indexes how frequently this pair of identities was sorted into the same cluster in the specific listener group (0 = never, 1 = always). Perfect performance would be indicated by all within-talker pairs being 1 (i.e., dark shading in the upper left-hand quarter and lower right-hand quarters of the matrix, indexing listeners’ ability to “tell people together”), and all across-talker pairs being 0 (i.e., very light shading in the upper right-hand quarter and lower left-hand quarters of the matrix, indexing listeners’ ability to “tell people apart”) (Colour figure online)

As reported in previous voice and face identity sorting studies (Jenkins et al., 2011; Lavan, Burston, & Garrido, 2019a; Stevenage et al., 2020), sizeable effects of talker familiarity are immediately apparent in our cluster, “telling together”, and “telling apart” data (see Figs. 2 and 3). Following Bonferroni correction for 4 pairwise comparisons within each dependent variable, only p-values under .0125 are reported as significant.

Listeners who were familiar with the talkers through having watched the TV show formed significantly fewer clusters than listeners who were not familiar with the talkers, for both accent familiarity groups. Specifically, the Talker_HI_Accent_HI_ group formed significantly fewer clusters (Median = 3 clusters, range: 2–10) than the Talker_LO_Accent_HI_ group (Median = 5.5 clusters, range: 2–10, *W* = 191, *p* < .001, *r* = .55). Similarly, the Talker_HI_Accent_LO_ group formed fewer clusters (Median = 4 clusters, range: 2-9) than the Talker_LO_Accent_LO_ group (Median number = 5 clusters, range: 3–11; *W* = 224, *p* < .001, *r* = .45).

Clear effects of talker familiarity are also apparent in the “telling together” scores: Listeners who were familiar with the talkers performed significantly better at “telling people together” than listeners who were not familiar with the talkers, for both accent groups. Specifically, the Talker_HI_Accent_HI_ group (Median = 0.82, range: .23–1.0) performed significantly better than the Talker_LO_Accent_HI_ group (Median = 0.34, range: .12–1.0; *W* = 887, *p* < .001, *r* = .63). Similarly, the Talker_HI_Accent_LO_ group (Median = 0.50, range: .18–.96) performed significantly better than the Talker_LO_Accent_LO_ group (Median = 0.23, range: .12–.56; *W* = 860, *p* < .001, *r =* .74).

For “telling people apart,” there was a talker familiarity effect only in the presence of higher accent familiarity. Specifically, the Talker_HI_Accent_HI_ group (Median = 0.01, range: 0–0.35) performed significantly better than the Talker_LO_Accent_HI_ group (Median = 0.11, range: 0–0.41; W = 316, *p* = .008, *r* = .33). However, “telling apart” performance was similar for the Talker_HI_Accent_LO_ (Median = 0.16, range 0–0.36) and Talker_LO_Accent_LO_ groups (Median 0.16, range: 0–.34]; *W* = 471, *p* = .902, *r* = .02).

### Effects of accent familiarity

Effects of accent familiarity were overall smaller, but also more variable than the talker familiarity effects. Following Bonferroni correction for 4 pairwise comparisons within each dependent variable, only p-values under .0125 are reported as significant.

There was no significant effect of accent familiarity on the number of clusters formed by listeners. Specifically, there was no difference between the Talker_HI_Accent_HI_ group (Median = 3 clusters, range: 2–10) and the Talker_HI_Accent_LO_ group (Median = 4 clusters, range: 2–9; *W* = 579, *p* = .077, *r* = .23) nor between the Talker_LO_Accent_HI_ group (Median = 5.5 clusters, range: 2–10) and the Talker_LO_Accent_LO_ group (Median = 5 clusters, range: 3–11; *W* = 510.5, *p* = .821, *r* = .03).

Performance on “telling people together” showed variable effects of accent familiarity in terms of how the clusters were formed. Despite consistent numerical advantages, a significant accent familiarity effect was only observed in the presence of high talker familiarity. Specifically, the Talker_HI_Accent_HI_ group (Median “telling together” 0.82, range: .23–1.0) performed significantly better than the Talker_HI_Accent_LO_ group (Median 0.50, range: .18–.96; *W* = 236, *p* = .001, *r* = .41) but there was no significant difference between the Talker_LO_Accent_HI_ group (Median 0.34, range: .12–1.0) and the Talker_LO_Accent_LO_ group (Median 0.23, range: .12–.56; *W* = 362, *p* = .029, *r* = .27).

For “telling apart,” we again only found a significant accent familiarity effect in the presence of higher talker familiarity. Specifically, the Talker_HI_Accent_HI_ group (Median “telling apart” = 0.01, range: 0–0.35) performed significantly better than the Talker_HI_Accent_LO_ group (Median = 0.16, range: 0–.36; *W* = 616, *p* = .011, *r* = .33), but there was no significant difference between the Talker_LO_Accent_HI_ group (Median = 0.11, range: 0–.41) and the Talker_LO_Accent_LO_ group (Median = 0.16, range: 0–.34; *W* = 690, *p* = .034, *r* = .26).

### Summary

Our results suggest that both talker and accent familiarity can benefit performance on a voice identity sorting task. We observed medium-to-large talker familiarity effects for the number of clusters formed and “telling together” scores, while for “telling apart” a medium-sized effect was seen only in the presence of high accent familiarity. Effects of accent familiarity were, in contrast, mostly small and nonsignificant. We found medium-sized, significant accent familiarity benefits for “telling together” and “telling apart,” but this time only in the presence of high talker familiarity. This pattern of results in fact reveals that the most comprehensive performance advantages are found in the Talker_HI_Accent_HI_ listeners, relative to listeners who lack one or other type of familiarity. It appears there is therefore a hierarchy of effects: not only are talker familiarity benefits larger and more widespread, but accent familiarity effects appear to be dependent on talker familiarity to become manifest in our task.

## Discussion

We have conducted, to our knowledge, the first examination of both talker and accent familiarity on voice identity sorting behaviour. In line with our predictions, we find that although both types of familiarity can affect performance, the benefits of talker familiarity are larger and more consistent across performance measures. We discuss the results in detail below.

Familiarity with the talkers, acquired by having watched the TV show in which they featured, had substantial effects on voice identity sorting performance, as also demonstrated in previous studies (Lavan, Burston, & Garrido, 2019a; Lavan, Burston, Ladwa, et al., 2019b; Lavan, Kreitewolf, et al., 2021b; Lavan et al., 2016; Stevenage et al., 2020). Specifically, listeners who were familiar with the talkers outperformed listeners who were not familiar with the talkers by perceiving significantly fewer*—*and closer to the veridical number of 2—identities in our voice sorting task. This pattern of results was also apparent for listeners’ “telling together” scores, showing that listeners who were familiar with the talkers perceived naturally varying recordings of a talker as coming from the same identity, while listeners who were unfamiliar with the talkers were more likely to perceive recordings of the same talker as coming from several different identities. Finally, talker familiarity effects were also apparent for “telling apart,” but only for the listeners who were familiar with the accent.

Intriguingly, accent familiarity had no effect on the number of talkers perceived: Listeners from Northern Ireland (Accent_HI_) and England (Accent_LO_) formed a similar number of clusters. The number of perceived talkers (as measured by counting the number of clusters formed) may in the end be a relatively crude measure of accuracy in voice sorting studies: Each cluster*—*be it large or small*—*is treated equivalently by this measure, while the accuracy of the composition of the cluster (e.g., does a cluster include recordings from only one voice or were the identities erroneously mixed?) is not taken into account. This lack of sensitivity may have obscured the effects of accent. Indeed, our previous study measuring within-subjects effects of vocal expressiveness on voice identity sorting found no significant difference in the number of clusters formed for high-expressive versus low-expressive stimuli (Lavan, Burston, Ladwa, et al., 2019b). However, when looking at potentially more sensitive measures indexing how the different clusters were composed, accent familiarity effects did emerge in the present dataset. Specifically, listeners from Northern Ireland, who were highly familiar with the accent *and the talkers* used in the task, were better able to accurately sort multiple recordings of the same identity into the same cluster (“telling together”), while also making fewer “telling apart” errors (i.e., mixing talkers within clusters). Our findings therefore align with those previously reporting benefits of accent familiarity for voice identity task performance (Braun et al., 2018; Stevenage et al., 2011), as well as those that argue these effects can be small and inconsistent (e.g., Johnson et al., 2018; Yu et al., 2021).

We additionally predicted that effects of talker familiarity would be larger than effects of accent familiarity. Following an approach taken by Perrachione (2018), we qualitatively compared effect sizes in order to interpret the relative benefits of talker and accent familiarity to voice identity sorting performance. Effects for contrasts of talker familiarity were medium to large, while effects for contrasts of accent familiarity were small to medium. As outlined in the Introduction, these differences in the size of familiarity effects are to some extent intuitive: Knowledge about how a specific individual talker’s voice sounds is directly relevant to making identity judgements on stimuli produced by that individual. Indeed, being familiar with a talker may immediately permit recognition (“this is a voice I know”) and identification (“it’s Amir”) when that talker’s voice is heard. In contrast, accent familiarity alone can never trigger person recognition/identification*—*being familiar with a Glasgow accent, and even speaking with this accent oneself, does not permit recognition or identification of individuals without further information (“Hi. I’m Amir”). By this account, accent familiarity is conceptually secondary to talker familiarity, in that knowing an accent can assist the performance of voice recognition or identification, but knowing the talker is essential for these to succeed; this is reflected in our observed effect sizes. However, we must note that not all voice identity tasks demand recognition or identification. Accent and talker familiarity may have more comparable effects on a task like voice identity discrimination, which forces listeners to closely compare voice clips one pair at a time and which can be performed well without any prior talker familiarity. Indeed, in recent work we found that unfamiliar and familiar listeners use the acoustic distance between voice clips similarly when making same/different judgements in a voice identity discrimination task (Lavan, Kreitewolf, et al., 2021b). Given the self-directed nature of voice identity sorting tasks, where we typically place no limit on the number of times individual clips can be listened to and compared, we cannot tell whether listeners were forming their identity clusters primarily via recognition (“I know this talker”), identification (“It’s Michelle”), discrimination (“This voice is different to that one”), or some combination of these. Nonetheless, knowing a talker can enhance success with all of these possible strategies, while accent familiarity alone can only assist with one.

Further inspection of our results supports this suggested hierarchy in the different familiarity effects, where talker familiarity exceeds accent familiarity in the size of its influence on task performance. Specifically, beneficial accent familiarity effects on voice identity sorting were only statistically significant for “telling together” and “telling apart” when comparing the groups of listeners who were already familiar with the talkers (i.e., Talker_HI_ listeners). We did not set out to test for an interactive effect in this study, and as noted above the definition of participant groups does not allow for a clean orthogonality of the two familiarity types. Furthermore, we could not explicitly examine an interaction effect statistically due to the highly non-normal nature of sorting task data, which should be analyzed using nonparametric approaches. However, the patterns we have observed do suggest, as discussed above, that talker familiarity in this study is a prerequisite for significant accent familiarity benefits. Even more strikingly, this is the case despite the fact that the difference in (self-reported) accent familiarity between our Talker_HI_Accent_HI_ and Talker_HI_Accent_LO_ groups was overall smaller (mean ratings of 9.91 vs. 4.79, respectively) than between the Talker_LO_Accent_HI_ and Talker_LO_Accent_LO_ groups (mean ratings of 9.34 vs. 2.70, respectively). The relatively higher self-reported familiarity of the Talker_HI_Accent_LO_ listeners compared with the Talker_LO_Accent_LO_ group comes from an unavoidable aspect of our experimental design—listeners who were familiar with the talkers from the TV show *Derry Girls* had been, by definition, actively engaged with listening to the numerous Northern-Irish-accented characters who featured in the show. However, this limitation of the design makes it even more striking that it was in the Talker_HI_ listeners that accent familiarity was seen to be more beneficial to voice sorting task performance. This further underpins the argument that accent familiarity can build on talker familiarity for voice identity sorting, but not the other way around.

A novel finding of the current study lies with the pattern of results for “telling apart.” Both for voice identity sorting and face identity sorting, “telling apart” scores usually show very low error rates, regardless of familiarity with the voices/faces. That is, when forming clusters to represent individual identities, unfamiliar and familiar viewers and listeners rarely combine 2 different identities into the same perceived identity cluster (for voices, see J. Johnson et al., 2020; Lavan, Burston, & Garrido, 2019a; Lavan, Burston, Ladwa, et al., 2019b; Lavan, Collins, & Miah, 2021a; Lavan, Smith, & McGettigan, 2022; Stevenage et al., 2020; for faces, see Jenkins et al., 2011; J. Johnson et al., 2018; Lavan, Collins, & Miah, 2021a, Lavan, Smith, & McGettigan, 2022; Redfern & Benton, 2017). Where we have previously seen increased “telling apart” errors in voice sorting studies, these have emerged in direct comparisons of different stimulus sets (i.e., highly expressive clips including whispering, shouting, and emotional speech vs. low expressive conversational speech; Lavan, Burston, Ladwa, et al., 2019b) or task instructions (i.e., when unfamiliar listeners have been instructed to sort the sounds into a two-identity solution; Lavan et al., 2020). In the current study, however, we found significant differences in “telling apart”—namely, that the Talker_HI_Accent_HI_ group performed better in all comparisons with other groups. Although “telling apart” scores should not be interpreted in an absolute sense, given that these could be affected by the choice of talkers and stimuli (e.g., some voice sorting tasks will feature more confusable talkers than others), it is striking that in the current experiment the Talker_HI_Accent_HI_ group produced a median of 1% “telling apart” errors, compared with 11%–16% in the other groups. Thus, whatever the combined benefits of talker and accent familiarity, the presence of both types of familiarity afforded listeners in the current study the ability to almost completely avoid confusing the two talkers with each other.

This difference in talker familiarity and accent familiarity effect sizes could suggest that accent and talker familiarity may be additive or interactive during identity perception. For example, in line with the theoretical explanations for language familiarity effects outlined by Perrachione (2018), listeners who were familiar with the accent could have accessed phonetic, phonological, and linguistic information in the stimuli that was less accessible to listeners less familiar with the accent. This additional information may also have enabled them to better access talker idiosyncrasies, improving their performance on voice identity sorting. A potential interaction may be implicated in the encoding of talker identity information when the Talker_Hi_Accent_HI_ listeners originally watched the TV show and were already better able to encode the verbal content than listeners who were less familiar with the accent. That is, accent knowledge may be used to form a talker representation and thus shape its contents. Our study, was, however, not explicitly designed to explore such interactions or indeed to identify the mechanisms through which accent familiarity advantages may arise. As such, further work will be required to tackle these questions.

Some authors have argued that social bias may play a role in several previous reports of accent familiarity effects (e.g., Braun et al., 2018; Yu et al., 2021). Previous work has reported the effects of such bias when listening to voices: For example, participants rated statements spoken in foreign accents to be less credible, even when asked to ignore the influence of speech intelligibility when making their judgements (Lev-Ari & Keysar, 2010). Listeners may therefore perform differently on voice identity tasks when speakers are recognized as belonging to social out-groups (Yu et al., 2021). A mechanism for how bias might impair voice identity processing is not clear, and future work is needed to disentangle the contributions of such higher-order factors from perceptual mechanisms of voice perception (Yu et al., 2021). It is furthermore unclear if, and to what extent, social bias could have affected performance in the current voice sorting task. By using only one set of stimuli and one accent, we to some extent avoided the possible pitfalls of within-subjects comparisons across accents that might have connoted differing stereotypes or socioeconomic statuses. However, while the Derry accent was primarily chosen because it is very rarely heard in Great Britain outside of the context of the *Derry Girls* show, we have noted that the voices chosen are depicted as working-class Catholics in Northern Ireland and thus are socially marked. To participants who had watched the show, these social factors may indeed be more apparent. However, both negative and positive social bias could be present across all our participant groups: English listeners may perceive Derry-accented speech to be socially distant because it sounds unfamiliar, while Northern Irish listeners from another city or social class to the characters might have different reasons to be negatively biased. On the other hand, participants who had watched *Derry Girls* and signed up for a study about it might be expected to feel favourably toward the voices because of their positive associations with a TV show they enjoyed watching. It would be valuable to explore the possible roles of broader social factors on task performance in future work.

We note a caveat regarding our discussion of effect sizes. One potential limitation in the current design is that, unlike in our previous work examining talker familiarity effects in voice sorting (Lavan, Burston, & Garrido, 2019a; Lavan, Burston, Ladwa, et al., 2019b), no participant was excluded based on their ability to remember the specific recordings used in the task. This was due to data collection errors, where questionnaire responses about self-reported memory for specific clips in the task were not obtained for some participants. Although we note that the talker familiarity effect sizes reported in the current study are broadly in line with other voice identity sorting studies measuring talker familiarity effects (see Lavan, Burston, & Garrido, 2019a; Lavan, Burton, Scott, & McGettigan, 2019c; Stevenage et al., 2020), the talker familiarity advantage may have been boosted due to a partial confound of talker familiarity with *stimulus* familiarity.

There are a number of ways in which future work could resolve some of the limitations of the current study. One such limitation was that our Talker_HI_Accent_LO_ reported greater familiarity with Northern Irish accents than the Talker_LO_Accent_LO_ group. Any participants with otherwise low exposure to Northern Irish accents in everyday life will tend to report a somewhat heightened sense of familiarity through watching *Derry Girls* and hearing the different characters within it. This unintended consequence of the design made it even more striking that accent familiarity effects on voice identity sorting performance were only seen for the Talker_HI_ participants in the current study, for whom the Accent_HI_ vs. Accent_LO_ differences in self-reported accent familiarity were smaller. Nonetheless it would be preferable to run another version of the study in which the low accent familiarity groups were better matched on this parameter. This could be done through testing additional listeners from the Talker_LO_Accent_LO_ participant pool and then selecting a final set for whom average accent familiarity matches the Talker_HI_Accent_LO_ group. This would of course be potentially wasteful of data due to the need to exclude participants for the matching process, and moreover would potentially place undue reliance on the precision of self-report scores to assess accent familiarity. Another consideration would be to test a new set of participants on recognition of talkers from *Derry Girls* when they speak in a different accent, in order to separate the accent in which the voices were learned from the accent heard at test. However, this would address a slightly different question about the generalization of identity perception across accents, and would present its own difficulties in terms of controlling accent familiarity (e.g., we cannot assume that listeners would be less familiar with the Republic of Ireland accents of the actors who portray Clare and Orla in *Derry Girls* than they would be with the Derry accents of Erin and Michelle).

A second major limitation of the current study is the possible confound of talker familiarity with stimulus familiarity, also discussed above. Perhaps the most promising way to address this in future work would be to use a voice sorting task including the same voices used in Experiment 1 but using clips from outside the *Derry Girls* show (e.g., excerpts from interviews, podcasts, and other media appearances). This is by no means a perfect control—despite playing roles in *Derry Girls* using their native Derry accent, the two actors are still portraying character voices with their own idiosyncrasies, who do not necessarily speak exactly as the actors would in everyday life. However, this approach to stimulus selection would greatly reduce the possibility that the clips would have been heard before, and consequently that episodic memory for specific utterances could be used in a task strategy.

Models of voice processing have proposed that the different processing pathways for speech, identity and emotion are largely independent*—*with some scope for interactions (Belin et al., 2004; Belin et al., 2011). Adding to pre-existing evidence from voice sorting studies that familiarity with specific talkers can improve identity perception in the presence of naturally varying stimuli, here we report new evidence that speech-relevant cues can, depending on their accessibility to the listener, additionally support identity processing. This aligns with previous work showing that interactions exist between the different processing pathways when listening to voices: For example, it has been shown that speech intelligibility in challenging listening situations is improved when a person is familiar with the talker (Holmes et al., 2018; Johnsrude et al., 2013; Kreitewolf et al., 2017; Nygaard & Pisoni, 1998). Together, these findings form a body of evidence that information that is diagnostic for the identity of a talker is not only encoded in the sound of their voice but also in *how they speak—*including cues that vary due to societal or geographic factors, as well as idiosyncrasies. It could be argued that, in naturalistic settings, the point at which speech perception ends and voice perception begins is therefore impossible to determine.

We therefore conclude that our study provides further evidence that voice identity perception is a multifaceted and possibly interactive process between aspects of voice and speech perception. During voice identity processing, listeners are likely to use any information available to them to advance their perceptual goals, irrespective of which processing pathway it may primarily belong to (Kreiman & Sidtis, 2011; Lavan & McGettigan, 2019). However, there is also a hierarchy of effects, where the beneficial effects of familiarity with a general speaking style (e.g., accent) are dependent on coexisting familiarity with the specific talkers being heard in the task. Future work is required to more fully explore when and how these different kinds of information are retrieved and combined to support aspects of voice processing.
